# Inhibition of ERRα suppresses epithelial mesenchymal transition of triple negative breast cancer cells by directly targeting fibronectin

**DOI:** 10.18632/oncotarget.4436

**Published:** 2015-06-26

**Authors:** Ying-Min Wu, Zhuo-Jia Chen, Hao Liu, Wei-Dong Wei, Lin-Lin Lu, Xiang-Ling Yang, Wei-Ting Liang, Tao Liu, Huan-Liang Liu, Jun Du, Hong-Sheng Wang

**Affiliations:** ^1^ Department of Microbial and Biochemical Pharmacy, School of Pharmaceutical Sciences, Sun Yat-sen University, Guangzhou 510006, China; ^2^ Sun Yat-sen University Cancer Center, State Key Laboratory of Oncology in South China, Collaborative Innovation Center for Cancer Medicine, Guangzhou 510060, China; ^3^ Cancer Research Institute and Cancer Hospital, Guangzhou Medical University, Guangzhou 510095, China; ^4^ Guangdong Provincial Key Laboratory of Colorectal and Pelvic Floor Diseases, Guangdong Institute of Gastroenterology and The Sixth Affiliated Hospital, Sun Yat-sen University, Guangzhou 510655, China; ^5^ Institute of Human Virology and Key Laboratory of Tropical Disease Control of Ministry of Education, Sun Yat-sen University, Guangzhou 510080, China

**Keywords:** ERRα, TNBC, EMT, fibronectin

## Abstract

Triple-negative breast cancer (TNBC) patients have poor prognosis due to the aggressive metastatic behaviors. Our study reveals that expression of estrogen related receptor α (ERRα) is significantly (*p* < 0.01) positively associated with high grade tumors and lymph node metastasis, while negatively correlated with overall survival (OS), in 138 TNBC patients. Targeted inhibition of ERRα by its inverse agonist XCT-790 or si-RNA obviously inhibits *in vitro* motility of TNBC cells. While over expression of ERRα triggers the invasion and migration of TNBC cells. Further, si-ERRα and XCT-790 inhibit the epithelial mesenchymal transition (EMT) of TNBC cells with increasing the expression of E-cadherin and decreasing fibronectin (FN) and vimentin. While XCT-790 has no effect on the expression of EMT related transcription factors such as Snail or Slug. Further, inhibitors of MAPK, PI3K/Akt, NF-κB signal molecules, which are activated by XCT-790, can not attenuate the suppression effects of XCT-790 on EMT. Alternatively, luciferase reporter gene assays and ChIP analysis indicate that ERRα can directly bind with *FN* promoter at ERR response element-3 (ERRE-1), ERRE-3, and ERRE-4, while XCT-790 reduces this bond. *In vivo* data show that ERRα expression is significantly (*p* < 0.05) correlated with FN in clinical TNBC patients. In MDA-MB-231 tumor xenograft models, XCT-790 decreases the expression of FN, inhibits the growth and lung metastasis, and suppresses the EMT. Our results demonstrate that ERRα functions as a metastasis stimulator and its targeted inhibition may be a new therapeutic strategy for TNBC treatment.

## INTRODUCTION

Triple-negative breast cancer (TNBC) is defined by lack of expression of estrogen receptor (ER), progesterone receptor (PGR) and human epidermal growth factor receptor 2 (HER-2) [1]. Since the lack of common therapeutic targets, TNBCs are neither susceptible to endocrine therapy (Tamoxifen and aromatase inhibitors) nor to targeted therapeutics (Trastuzumab and Lapatinib) [1]. Further, TNBCs are associated with increased risks of metastasis and high rates of recurrence. So far there is no FDA (Food and Drug Administration)-approved targeted therapy for TNBC patients. Therefore, TNBC patients have the worst prognosis and shortest survival rates among all subtypes of breast cancer [2]. There is an urgent need to develop targeted therapy approaches for TNBC treatment.

Epithelial–mesenchymal transition (EMT) is a developmental process in which epithelial cells lose polarity and develop a mesenchymal phenotype [3]. It has been considered as the initiation process of cancer metastasis including TNBC [3]. Tumor occurring EMT can acquire invasive mesenchymal phenotypes, increase motility and invasiveness, and infiltrate into the tumor vascular. The EMT is positively correlated with tumor progression, maintenance, drug resistance, and metastasis [4, 5]. The progression of EMT is promoted by several important transcription factors such as Snail, Slug, Twist and Zeb [6]. Activation of multiple cellular signal pathways such as MAPK, PI3K, NF-κB, and Wnt/β-catenin also can trigger the EMT process [5, 7]. Increasing evidences show that the progression of TNBC, the most aggressive metastatic behavior of breast cancer subtype, is associated with the process of EMT [8, 9]. Further, EMT related signal pathways such as MAPK and PI3K/Akt are highly activated in TNBC cells [10]. Therefore the inhibition of EMT might be a potential strategy to improve the prognosis of TNBC patients.

The estrogen-related receptor alpha (ERRα) is an orphan member of the superfamily of nuclear hormone receptors [11]. It has similar structure with estrogen receptor (ER) and controls the expression of genes related to cell metabolism such as tricarboxylic acid cycle [12] and aerobic glycolysis [13, 14]. Levels of ERRα have been reported to be elevated in the more-aggressive tumors in breast cancer [15–17] as well as other types of tumors [18], which are associated with a worse prognosis. XCT-790, the specific inverse agonist of ERRα, can inhibit the proliferation of breast cancer cells [19, 20]. The ablation of ERRα in knock-out mice delays ERBB2-induced mammary gland tumorigenesis [21]. Further, studies revealed that inactivation of ERRα impairs *in vitro* migration of breast cancer cells [17, 22], while over expression of ERRα in xenografted breast cancer cells increases their metastatic capacities by induction of tumoral angiogenesis and up regulation of VEGF [23–25]. However, the roles of ERRα in TNBC progression and whether it is related to EMT process are still not studied.

In the present study, we show that inactivation of ERRα suppresses the migration and invasion of TNBC cells via inhibition the process of EMT both *in vitro* and *in vivo*. At the molecular level, we demonstrate that inactivation of ERRα decreases its binding affinity with the promoter of fibronectin (FN) and then down regulates the transcription of FN. Therefore, our findings suggest that inhibition of ERRα might represent a novel targeted therapy in TNBC metastasis.

## RESULTS

### Expression of ERRα is negatively correlated with prognosis in TNBC patients

To date there were very limited data concerning the relationship of ERRα expression with prognosis of TNBC patients, we then performed immunohistochemistry for ERRα in a set of 138 TNBC tumors. Table 1 shows that elevated ERRα is significantly associated with high grade tumors (*p* = 0.035) and lymph node metastasis (*p* < 0.001) of TNBC. Kaplan-Meier analysis of all 138 patients demonstrated a statistically significant negative correlation between overall survival (OS) and ERRα expression level (*p* < 0.001). Further, statistical comparison of survival between groups with the log-rank statistic analysis suggested that patients whose tumors express increased levels of ERRα had poorer survival compared with those with low levels of ERRα (*p* < 0.001) (Supplementary Figure S1). These data suggested that increased expression of ERRα resulted in a more aggressive phenotype in TNBC patients.

**Table 1:** ERRα expression in 138 TNBC patients

Characteristics	*N*	ERRα Low/No	ERRα High/Medium	*p* value
**Age**				
≤ 50	66	37	29	0.582
> 50	72	37	35	
**Stage**				
I/II	87	50	37	0.236
III/IV	51	24	27	
**Grade**				
I/II	54	35	19	0.035
III	84	39	45	
**Node metastasis**				
Negative (< 10)	81	57	24	< 0.001
Positive (≥ 10)	57	17	40	

### ERRα facilitates the *in vitro* motility of TNBC cells

**Figure 1:**
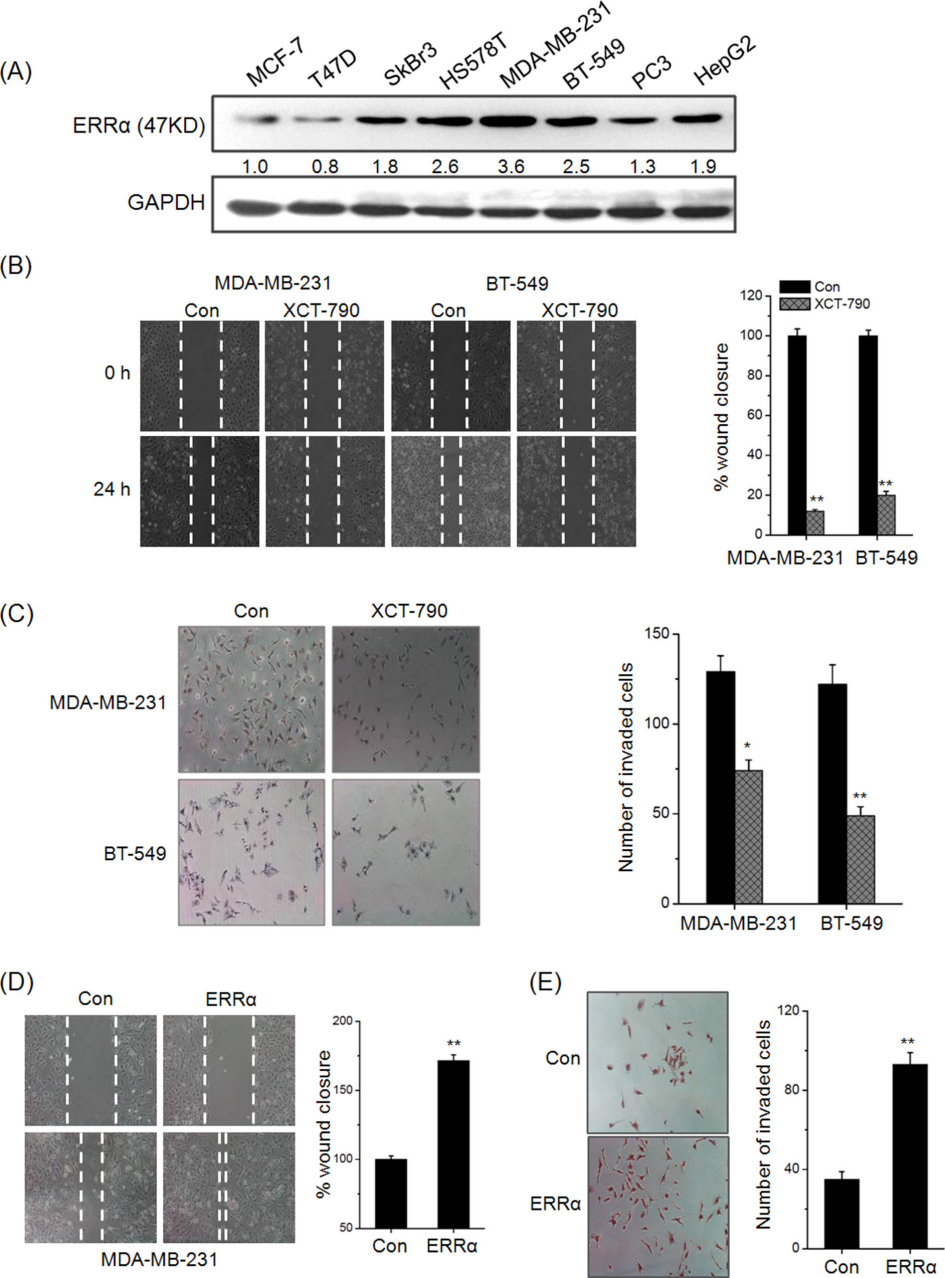
ERRα triggers the *in vitro* migration and invasion of TNBC cells **A.** The expression of ERRα in human cancer cells were measured by Western blot analysis; **B.** Confluent monolayers of MDA-MB-231 and BT-549 cells were scraped by a pipette tip to generate wounds and then treated with 5 μM XCT-790, respectively; **C.** MDA-MB-231 and BT549 cells were allowed to invade transwell chambers for 48 h in the presence or absence of 5 μM XCT-790. Then invaded cells were fixed, stained, and photographed; MDA-MB-231 cells were transfected with empty vector pcDNA3.1 or ERRα construct for 24 h, and then the cell motility was evaluated by wound-healing **D.** and transwell invasion assay **E.** Data represent the average of five independent experiments.

Clinical data revealed that elevated ERRα is significantly associated with lymph node metastasis, then we investigated the roles of ERRα in the motility of TNBC cells. As shown in Figure 1A, the expression of ERRα was low in MCF-7 and T47D cells, which have little metastatic powers, while was relatively high in MDA-MB-231, BT-549 and HS578T cells, which are capable of metastasizing. Then the roles ERRα on motility of TNBC cells were further investigated by use of *in vitro* wound-healing and transwell invasion assay. As shown in Figure 1B, treatment with 1 μM XCT-790 for 24 h obviously inhibited wound closure of both MDA-MB-231 and BT549 cells as compared to the control group. Further, the number of invaded MDA-MB-231 and BT549 cells treated with 1 μM XCT-790 for 48 h was significantly (*p* < 0.05) less than that of control cells (Figure 1C). In MDA-MB-231 cells transfected with ERRα construct for 24 h, the wound closure (Figure 1D) and invaded cells (Figure 1E) were significantly (*p* < 0.05) increased as compared to the control group. To further verify the role of ERRα inhibition on cell motility, we knocked know ERRα in MDA-MB-231 cells by it specific siRNA. The results showed that si-ERRα significantly inhibited wound closure and *in vitro* invasion of MDA-MB-231 cells (Supplementary Figure S2). Cell viability analysis revealed that these treatments had no significant (*p* > 0.05) effect on the proliferation of MDA-MB-231 and BT549 cells (data not shown). Collectively, our results revealed that ERRα can significantly trigger the motility of TNBC cells, its inhibition or knockdown can inhibit the migration and invasion of TNBC cells.

### Targeted inhibition of ERRα suppressed the EMT of TNBC cells

**Figure 2:**
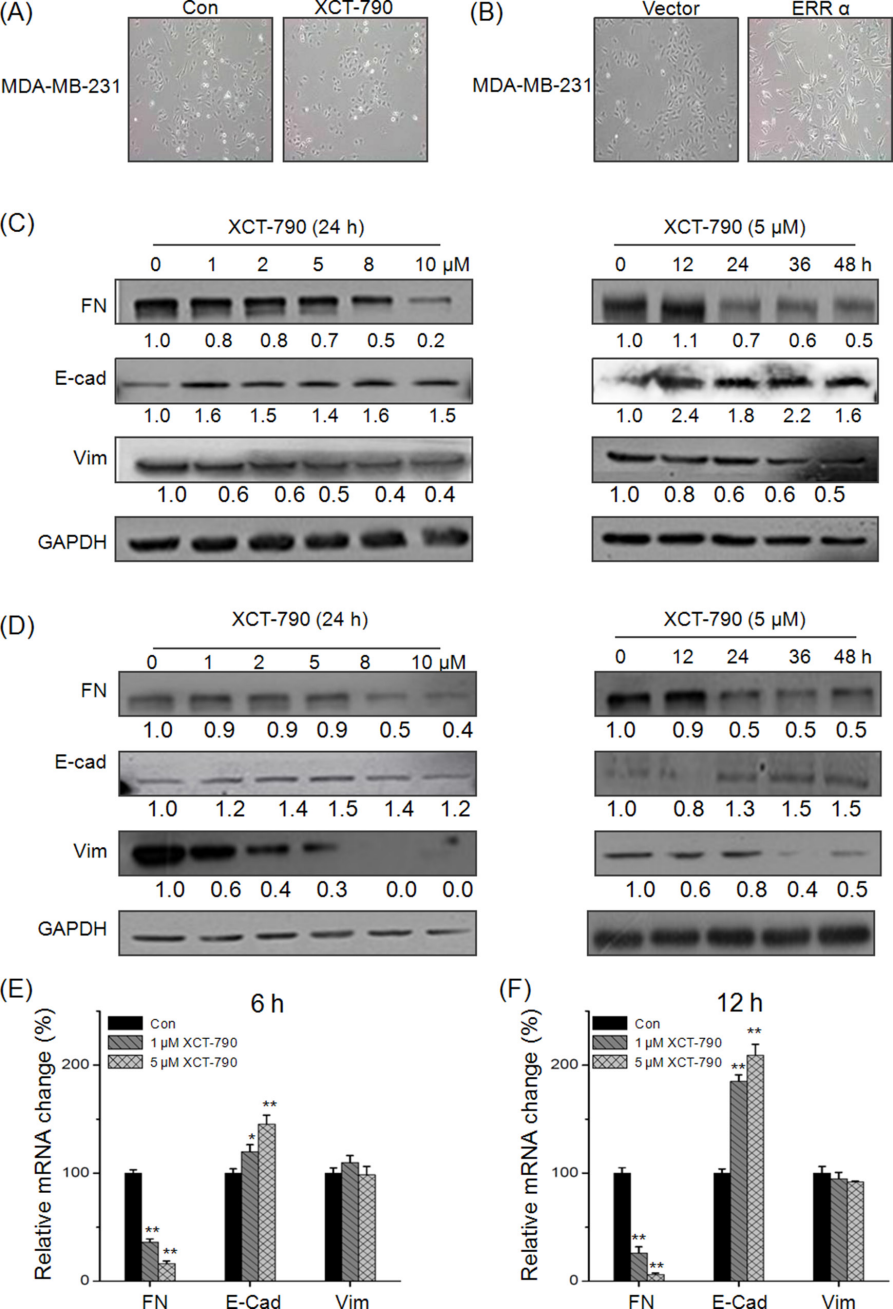
Targeted inhibition of ERRα suppresses the EMT of TNBC cells **A.** MDA-MB-231 cells were treated with or without 5 μM XCT-790 for 24 h; **B.** MDA-MB-231 cells were transfected with pcDNA3.1 (vector control) or ERRα construct for 24 h; MDA-MB-231 **C.** or BT-549 **D.** cells were treated with increasing concentration of XCT-790 for 24 h or 5 μM XCT-790 for the indicated times, and then the expression of FN, E-Cad, and Vim were measured by Western blot analysis; MDA-MB-231 **E.** or BT-549 **F.** cells were treated with 1 or 5 μM XCT-790 for 6 or 12 h, then the mRNA levels of FN, E-Cad, and Vim were measured by real-time PCR. Data represent the average of three independent experiments. *p < 0.05 compared with control, **p < 0.01 compared with control.

Increasing evidences show that the progression of TNBC is associated with the process of EMT [8, 9]. We then hypothesized that ERRα plays a positive role in the progression of EMT. Our results revealed that MDA-MB-231 cells treated with XCT-790 (Figure 2A) or transfected with si-ERRα (Supplementary Figure S3) lost their spindle-like fibroblast appearance and assumed a cobblestone-like epithelial morphology. Inversely, over expression of ERRα was associated with an increase of aggressive cell types (Figure 2B). This was also confirmed by Western blot analysis on XCT-790 treated MDA-MB-231 cells (Figure 2C) and BT-549 (Figure 2D) cells, which showed an increased expression of the epithelial cell marker E-Cadherin (E-cad), and an decreased expression of the mesenchymal cell markers FN and vimentin (Vim) via both time and concentration dependent manners. Furthermore, qRT-PCRs analysis showed that XCT-790 treatment down regulated FN while up regulated E-Cad at mRNA levels in both MDA-MB-231 (Figure 2E and 2F) and BT-549 (Supplementary Figure S4) cells via both time and concentration dependent manners, while had limited effects on the mRNA of Vim in these two cell lines. This was also confirmed by the results that si-ERRα obviously prevent (Supplementary Figure S5) while over expression ERRα significantly promote (Supplementary Figure S6) the EMT in MDA-MB-231 cells. Collectively, these observations showed ERRα can trigger the progression of EMT in TNBC cells, while targeted inhibition of ERRα by XCT-790 suppresses this process.

### EMT related transcription factors and signal pathways including MAPK, PI3K/Akt, NF-κB do not mediate the suppression effects of XCT-790 on EMT

**Figure 3:**
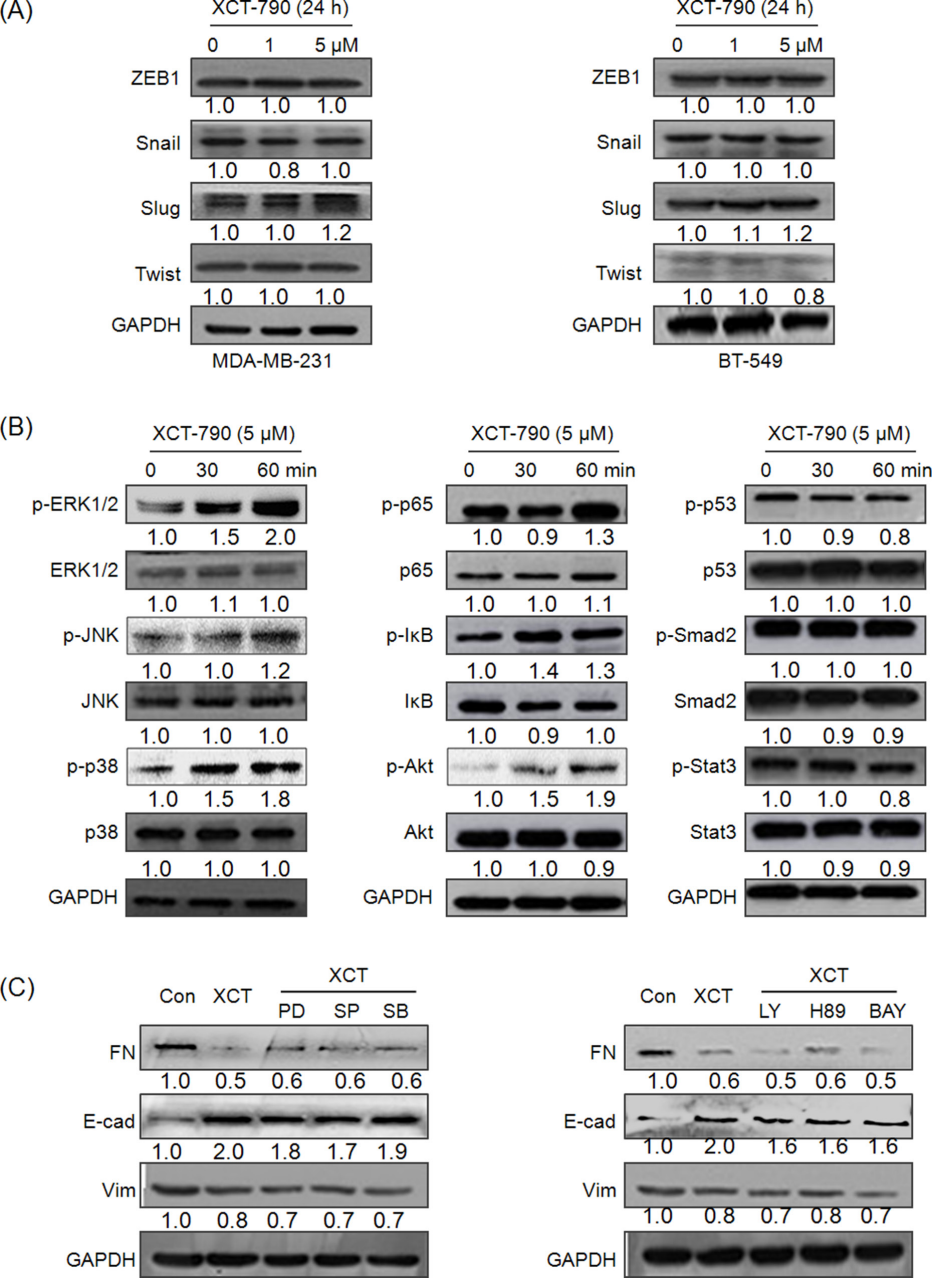
EMT related transcription factors and signal pathways including MAPK, PI3K/Akt, NF-κB do not mediate the suppression effects of XCT-790 on EMT MDA-MB-231 **A.** and BT549 **B.** were treated with 1 or 5 μM XCT-790 for 24 h, then the protein levels of Snail, Slug, Twist, and ZEB were detected by Western blot analysis; (B) MDA-MB-231 cells were treated with 5 μM XCT-790 for the indicated times, the phosphorylation and total levels of signal pathways including MAPK, PI3K/Akt, NF-κB, p53, Smad, and Stat3 were measured by Western blot analysis; **C.** MDA-MB-231 cells were pretreated 10 μM MEK inhibitor PD98059(PD), JNK inhibitor SP600125 (SP), p38-MAPK inhibitor SB203580 (SB), PI3K inhibitor LY294002 (LY), PKA inhibitor H-89, or NF-κB inhibitor BAY11–7082 (BAY) for 90 min, and then exposed to 5 μM XCT-790 for further 24 h, the expression of FN, E-Cad, and Vim were measured by Western blot analysis. Data represent the average of three independent experiments.

Since transcription factors Snail, ZEB1, Twist and Slug play essential roles in regulating EMT [4], we then investigated whether their expressions were altered in TNBC cells treated with XCT-790. Our results showed that XCT-790 treatment had no significant effect on the protein expression of Snail, Slug, Twist or ZEB1 in either MDA-MB-231 or BT-549 cells for various concentrations (Figure 3A). Cellular signal pathways including MAPK, PI3K/Akt, NF-κB, p53, Smad, and Stat3 are suggested to promote the progression of EMT in cancer cells [5]. Then their statuses in MDA-MB-231 cells treated with 5 μM XCT-790 for 30 or 60 min were checked by Western blot analysis. The results showed that XCT-790 slightly increased the phosphorylation of ERK1/2, JNK, p38-MAPK, p65, and Akt, while had no obvious effect on other signalling molecules such as p53, Smad2, or Stat3 (Figure 3B). To verify whether ERK1/2, JNK, p38-MAPK, p65, and Akt mediate the suppression effects of XCT-790 on EMT, we pretreated MDA-MB-231 cells with their inhibitor for 90 min and then exposed to XCT-790 for another 48 h. The results showed that inhibitors of ERK1/2, JNK, p38-MAPK, PI3K/Akt, PKA, or NF-κB can not attenuate XCT-790 induced FN down regulation and E-Cad up regulation (Figure 3C). Our data suggested that EMT related transcription factors and signal pathways including MAPK, PI3K/Akt, NF-κB do not mediate the suppression effects of XCT-790 on EMT.

### Targeted inhibition of ERRα directly inhibited the expression of FN

**Figure 4:**
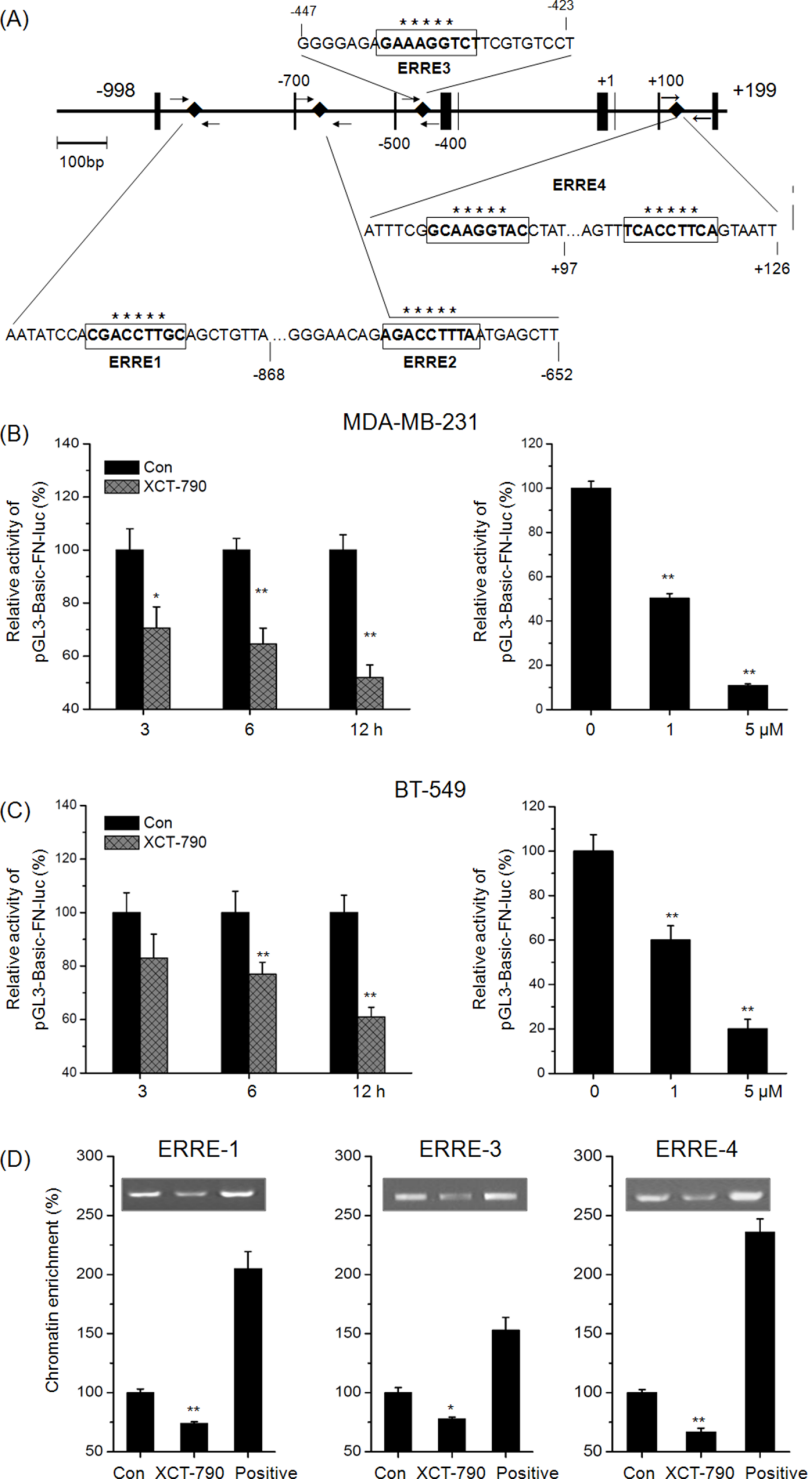
Targeted inhibition of ERRα directly inhibits the transcription of FN **A.** Diagram of the human FN gene locus showing ERRE-1~4 (boxed sequences) in the promoter. Small arrows indicate the regions amplified in the ChIP experiments. MDA-MB-231 **B.** or BT-549 **C.** cells were transfected with pGL3-Basic-FN-luc reporter plasmid containing FN promoter and then treated with or without XCT-790 (left column: 1 μM) for the indicated times (right column: 24 h). Luminescence was measured by a luminometer. pRL-TK plasmids served as the correcting transfection efficiency. Results were expressed as the ratios between the activity of the reporter plasmid and pRL-TK. MDA-MB-231 cells were treated with or without 5 μM XCT-790 for 24 h, and then the recruitment of ERRα to ERREs of FN promoter was determined by ChIP. Immunoprecipitated products were amplified by qPCR, using primers indicated in Supplementary Table S1. Data are expressed relative to the amount of DNA immunoprecipitated in control cells and are the mean ± SEM of 3 experiments. *p < 0.05 compared with control, **p < 0.01 compared with control.

Since transcription factors and the above signal pathways did not mediated the suppression effects of XCT-790 on EMT, we then investigated whether EMT biomarkers were directly regulated by ERRα. We analyzed the human *FN* and *CDH1* (E-Cad gene) promoter sequence *in silico* to detect potential ERR response element (ERRE) composed of the extended ERE half-site AAGGT (or its reverse complement ACCTT) [18, 26]. As show in Figure 4A, four potential ERREs were found located within the proximal promoter of *FN*. Then we transfected the promoter reporter gene plasmid pGL3-Basic-FN-luc into MDA-MB-231 and BT-549 cells and then treated with XCT-790 for the indicated times or concentrations. Our data showed that XCT-790 significantly decreased the activity of pGL3-Basic-FN-luc in MDA-MB-231 (Figure 4B) and BT-549 (Figure 4C) cells via both time and concentration dependent manners. It suggested that XCT-790 can inactivate the promoter of FN thereby decreasing the transcription of FN.

We next performed a ChIP assay to test whether ERRα binds to *FN* promoter in the native chromatin environment of TNBC cells. MDA-MB-231 cells were treated with 5 μM XCT-790 for 24 h, results showed that ChIP with a human ERRα-specific antibody resulted in a significant decrease in the genomic fragment that contained the ERRE-1 (0.74 fold), ERRE-3 (0.78-fold), and ERRE-4 (0.67-fold) sites in the *FN* promoter. As negative control, weak precipitation was detected with the use of no antibody or anti-rabbit IgG. Together, these data suggested that XCT-790 can inhibit the bind between ERRα and *cis-*regulatory domain of the endogenous *FN* promoter and then suppress the transcription of FN.

### The expression of ERRα was positively correlated with FN *in vivo*

**Figure 5:**
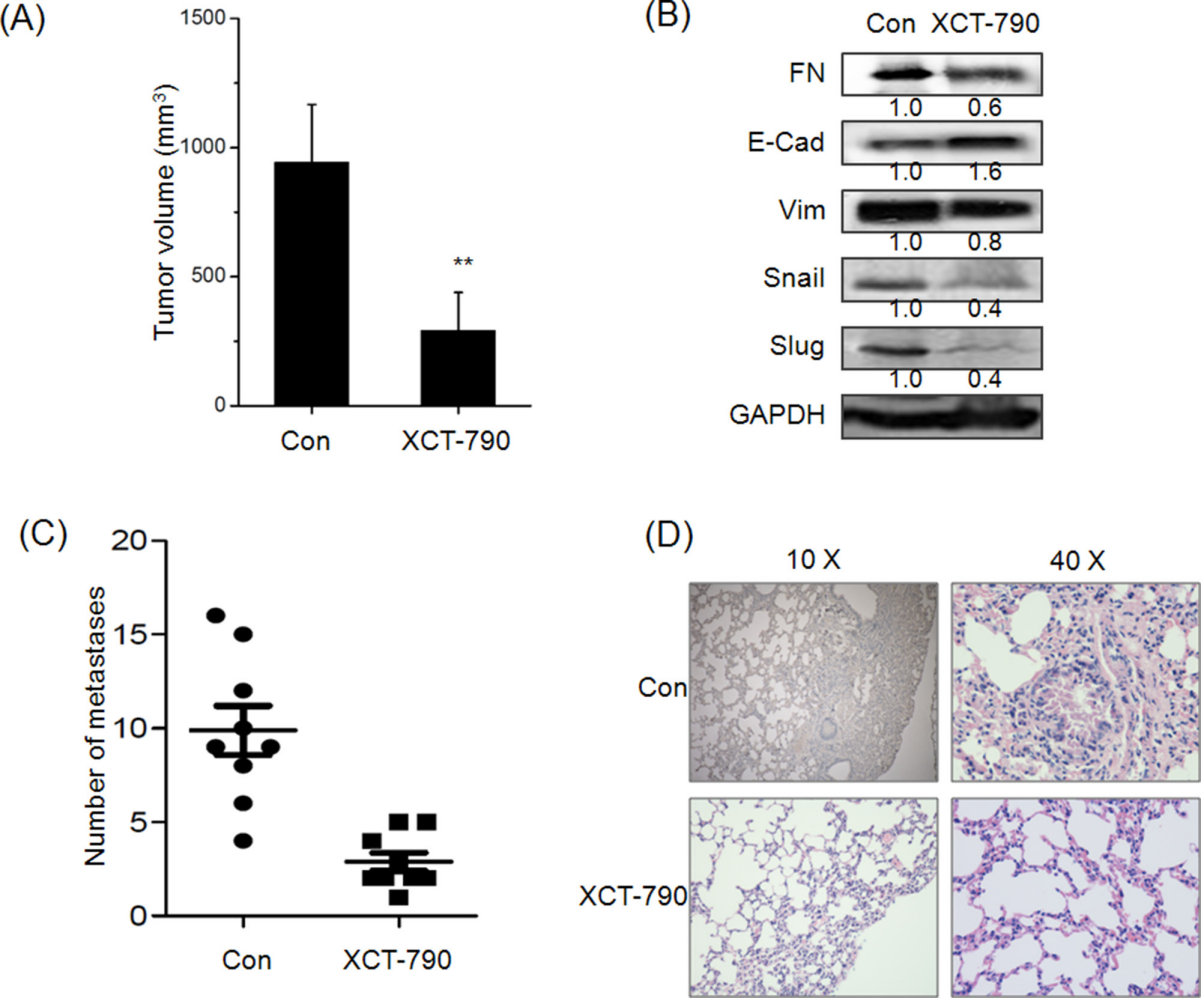
Targeted inhibition of ERRα inhibits the growth and metastasis of TNBC *in vivo* **A.** The tumor volume of XCT-790 and control group at the end of experiment; **B.** The EMT related biomarkers and transcription factors were determined by Western blot analysis in the tumor lysates from the control and XCT-790 treated group; **C.** Numbers of lung metastases in the control and XCT-790 treated group at the end of experiment; **D.** H&E examination of metastasis in lung tissue sections of the control and XCT-790 group. **p < 0.01 compared with control.

*In vitro* analysis showed that ERRα binds to *FN* promoter and then activates its expression directly. To evaluate the role of ERRα on *in vivo* FN expression, we checked the relationship between ERRα and FN expression in 138 TNBC tumors by immunohistochemistry. Of the 138 TNBC cases, 64 had elevated ERRα and 75 had low levels of FN. The Fisher's exact test showed that scores for ERRα and FN immunostaining were significantly correlated in 65.2% of tissues in this series (*p* < 0.001) (Table 2). This result suggested that ERRα expression is positively correlated with FN in clinical TNBC patients. We then examined the *in vivo* effect of ERRα on the FN expression in MDA-MB-231 tumor xenografts in nude mice by tail vein injection of XCT-790. Western blot analysis showed that XCT-790 treatment significantly decreased the expression of FN in primary tumors (Figure 5B). Generally, our data revealed that the expression of ERRα was positively correlated with FN *in vivo*, while its inhibition can suppress the FN expression.

**Table 2:** The expression of ERRα and FN is positively correlated in TNBC patients

	FN Low	FN Medium	FN high	Total
ERRα Low	63*	6	5	74
ERRα Medium	4	14*	6	24
ERRα High	8	10	22*	40
Total	75	30	33	138

* The number of cases in which ERRα and FN are correlated with each other.

### Targeted inhibition of ERRα inhibited the growth and metastasis of TNBC *in vivo*

As XCT-790 can suppress *in vitro* migration and invasion of TNBC cells, a key question was whether targeted inhibition of ERRα can inhibit metastatic behaviors *in vivo*. We then examined the effect of XCT-790 on metastasis of MDA-MB-231 tumor xenografts in nude mice. Our results revealed that the average size of tumor in XCT-790 group was significantly (*p* < 0.05) less than that of control ones at the end of *in vivo* experiment (Figure 5A). Further, Western blot analysis showed that XCT-790 treatment also significantly decreased the expression of Vim while up regulated E-Cad in primary tumors (Figure 5B). These data suggested that ERRα inhibition suppresses the metastasis in nude mice bearing MDA-MB-231 xenografts via EMT suppression of primary tumor. Interestingly, XCT-790 treatment also decreased the expression of Snail and Slug *in vivo*, which was contrary to *in vitro* results and needed further studies. Further, XCT-790 reduced the number of metastases to the lungs by 67%–85% (*p* < 0.05) compared with the control group (Figure 5C). This was confirmed by histological analysis which revealed that the extent of lung metastatic lesions derived from control group was greater than that of XCT-790 groups (Figure 5D). The data supported that targeted inhibition of ERRα suppresses the metastasis of TNBC *in vivo* by EMT suppression.

## DISCUSSION

Recent studies revealed that ERRα has a positive role in cancer cell migration and invasion [18, 21, 27]. One of the major characteristics of TNBC is aggressive migration and invasion, while the role and mechanism of ERRα on TNBC metastasis has not been well illustrated. Our present study reveals that targeted inhibition of ERRα can suppress the *in vitro* and *in vivo* motility of TNBC cells via EMT suppression by directly down regulating FN. Further, IHC results suggest elevated ERRα is significantly positively associated with FN expression, high grade tumors and lymph node metastasis, while negatively associated with overall survival of 138 TNBC patients. These results suggest ERRα activation as a mechanism of TNBC aggressiveness and imply that targeting ERRα may be a promising approach for TNBC treatment.

Our present study shows that targeted inhibition of ERRα suppresses the migration and invasion of TNBC both *in vitro* and *in vivo*. This is consistent with recent studies that ERRα can down regulate RHOA stability [27] or induce WNT11 expression [22] to increase cancer cell migration, while knockdown of ERRα during the early stages of zebrafish embryonic development results in inhibition of cell migration [28]. The expression of ERRα was also positively associated with markers of increased recurrence and adverse clinical outcome, such as increased expression of *MYC* oncogene, Ki-67 and AIB1 [16, 29, 30]. A molecular mechanism has been proposed linking ERRα to the activation of Wnt11-elicited pathway leading to increased N-cadherin expression [22], which is an important mesenchymal biomarker for cancer cells [31]. These data confirm that ERRα plays a positive role in metastasis of TNBC cells, while its inhibition can suppress the motility of TNBC cells.

Most TNBC patients exhibit gene expression patterns associated with EMT [32]. EMT can endow carcinoma cells with enhanced migratory and survival abilities and is considered as the a first and key step in cancer metastasis. When the EMT process is triggered, epithelial cells lose the expression of intercellular adhesive protein E-Cad and gain the mesenchymal markers such as FN and Vim [5]. Our present results reveal that inhibition of ERRα by XCT-790 or siRNA suppressed aspects of EMT in TNBC cells. While ectopic expression of ERRα promotes the EMT and induction of epithelial markers. All of these data confirm the positive role of ERRα in EMT of TNBC cells. During this process, Vim only changed in protein level rather than RNA level, which suggests that it may just play a accompany role. Similarly, Lam et al. also suggested that targeting ERRα inhibits EMT and stem cell properties of ovarian cancer cells [33]. As EMTs account for the aggressiveness and stemness of TNBC, targeting the EMT-like phenotype becomes a unique strategy for TNBC treatment. It is also worth noting that high levels of ERRα are found in various cancers such as colon, prostate, and ovarian cancers [18], suggesting that role of ERRα in EMT may have broader implications for other tumor cell types.

The mechanisms of ERRα in EMT progression are not well illustrated. Lam et al. suggested that targeted inhibition of ERRα inhibited the expression of Snail through both transcriptional and posttranscriptional regulation in ovarian cancer cells [33]. Inversely, inhibition of ERRα has no effect on EMT related transcription factors such as Snail, Slug, Twist, or ZEB. This might be due to the variation among different cancers. Further, although XCT-790 can slightly activate ERK1/2, JNK, p38-MAPK, p65, and Akt, their inhibitors have no effect on XCT-790 induced FN down regulation and E-Cad up regulation. As a transcription factor, our results indicate that XCT-790 can directly impair the bind between ERRα and promoter of *FN*, then decrease the transcription of FN (Figure 4). This was confirmed by the results that XCT-790 decreases the protein and mRNA levels and transcription activities of FN via both time and concentration dependent manners. The human *FN* promoter contains four ERREs which are suggested to be bound by ERRα, while ChIP results suggest that ERRE-1, ERRE-3, and ERRE-4 are the binding sites of ERRα in TNBC cells. Among the extracellular matrix (ECM) components, FN has often been used as one of the mesenchymal markers whose expression is strongly enhanced in EMT process [34]. Indeed, this is the first demonstration that ERRα is a directly acting transcription factor of the *FN* gene. Although we can not exclude additional mechanisms by which ERRα induces FN, our present study provides another mechanism for the positive role of ERRα in cancer metastasis.

ERRα over expression is correlated with poor outcome and bad prognosis in various cancer types such as prostate, colorectal, cervical and ovarian carcinomas [35]. In support of such a role in TNBC patients, a survey of 138 clinical TNBC samples shows significant correlations between increased ERRα levels and higher breast cancer grade, metastasis, and unfavorable outcome. In addition, the expression of ERRα and FN is significant positively correlated with each other in 65.2% TNBC patients. This suggests that over expression of ERRα might trigger the metastasis of TNBC cells via promoter EMT process in human body through up regulation of FN. Our study also revealed that ERRα has the potential to be used as a marker of unfavorable prognosis of TNBC.

Our findings are important for several reasons. We show that ERRα acts as mesenchymal function, which provides another mechanism for the aggressive phenotype of TNBC. We also present for the first time that ERRα can directly bind to the promoter of *FN* and then increase its expression. Further, ERRα can be an independent prognostic marker and a unique therapeutic target, with particular relevance to clinically aggressive TNBC. In summary, our results, taken together with published literatures, establish an essential role of ERRα in TNBC progression and position it as an important target for TNBC treatment. Considering that there is no efficiency therapy target for TNBC, our findings are encouraging and suggest that ERRα could be targeted for TNBC treatment in the future.

## MATERIALS AND METHODS

### Reagents

PD 98059 (PD, MAPK/ERK kinase inhibitor), SP600125 (SP, JNK inhibitor), SB203580 (SB, p38-MAPK inhibitor), LY294002 (LY, PI3K/Akt inhibitor), H89 (PKA inhibitor), BAY11-7082 (BAY, NF-κB inhibitor) were obtained from Sigma-Aldrich (St Louis, Mo., USA). XCT-790 (specific inverse agonist of ERRα) and other chemicals were of reagent grade or better and purchased from Sigma Chemical Co. (St. Louis, MO, USA) unless otherwise noted. Monoclonal antibodies against FN, E-Cad, Vim, Snail, Slug, Twist, ZEB, p-ERK1/2, ERK1/2, p-JNK, JNK, p-p38 MAPK, p38 MAPK, p-p65, p65, p-IκB, IκB, p-p53, p53, p-Smad2, Smad2, p-Stat3, Stat3, and GAPDH were purchased from Cell Signaling Technology Inc. (Beverly, MA, USA). Antibodies against p-Akt and Akt were purchased from Bioworld Technology, Inc (Minneapolis, MN, USA). Horseradish peroxidase-conjugated secondary antibody from Santa Cruz Biotechnology (Santa Cruz, CA, USA). All compounds were solubilized in DMSO. Medium containing 0.5% DMSO was used as the control.

### Cell culture and transfection

Cancer cell MCF-7, T47D, SkBr3, HS578T, MDA-MB-231, BT-549, PC3, HepG2, and SkBr3 cells were purchased from the American Type Culture Collection (Manassas, VA, USA), maintained in our laboratory, and cultured in RPMI 1640 or DMEM medium (Invitrogen Corporation, Carlsbad, CA, USA) supplemented with 10% heat-inactivated fetal Bovin serum, 100 U/ml penicillin, and 10 μg/ml streptomycin at 37°C in a 5% CO_2_ atmosphere. An ABI 3130 Genetic Analyzer (Applied Biosystems) was used for the profiling. The DNA profile data were cross-checked with the ATCC data bank. Medium was replaced with phenol red-free medium 24 h before experiments to remove the estrogen-like activity of phenol red. For transfection, cells were seeded into six-well plates and transfectd with pcDNA3.1 (vector control), ERRα construct (purchased from Addgene, Cambridge, MA), siRNA negative control (siRNA-NC: 5′-GGC TAC GTC CAG GAG CGC A-3′), or si-ERRα (sequence 5′-GAG CAU CCC AGG CUU CUC A dT dT-3′) by use of Lipofectamine 2000 reagent (Invitrogen).

### *In vitro* wound-healing and transwell invasion assay

A wound-healing assay was used to compare the migratory ability of MDA-MB-231 and BT549 cells as described previously [36]. The cell migration and invasion assay was using 6-well transwell plates (Falcon cell culture inserts, 8-μm pore size, BD, NJ) according to our previous study [37]. The *in vitro* wound-healing and transwell invasion assays were carried out at least 5 individual experiments.

### Western blotting analysis

Western blotting was performed as previously described [38].

### Quantitative real-time PCR

After treatment as indicated, total mRNA of cells was extracted with TRIZOL reagent. Then the quantitative real-time PCR for gene expression were conducted as our previous method [36]. Transcripts of the housekeeping gene GAPDH in the same incubations were used for internal normalization. Primer pairs were as follows: FN, forward 5′- CCC AGA CTT ATG GTG GCA ATT C-3′ and reverse 5′- AAT TTC CGC CTC GAG TCT GA-3′; E-Cad forward 5′- TAC ACT GCC CAG GAG CCA GA -3′ and reverse 5′- TGG CAC CAG TGT CCG GAT TA -3′; Vim forward 5′- TGA GTA CCG GAG ACA GGT GCA G -3′ and reverse 5′- TAG CAG CTT CAA CGG CAA AGT TC-3′; ERRα forward 5′-CA ATG AGT GTG AGA TCA CC -3′ and reverse 5′-CCG TTT GTA CTT CTG CCG TC-3′.

### Reporter genes assay

The pGL3-Basic-FN-luc which contains FN promoter with the sequence −998/+28 was the gift from Dr Tian Lan at Guangdong Pharmaceutical University. For measuring the transcriptional activity of FN, cells were transfected with 0.2 μg DNA/cm^2^ per plasmid and lipofectamine 2000 reagent (Invitrogen, USA) according to the manufacturer's instructions, and then treated with XCT-790 for the indicated times. Transfection efficiency was normalized by cotransfection with pRL-TK. Transcriptional activity was determined by a luminometer, using a dual-luciferase assay kit. Results were displayed as the ratios between the activity of the reporter plasmid and pRL-TK [37].

### Chromatin immunoprecipitation (ChIP)

ChIP assays were performed on MDA-MB-231 cells as previously described [20]. Briefly, cells were incubated for 10 min in phosphate-buffered saline containing 1% formaldehyde. After sonication with Bioruptor (Diagenode, Liège, Belgium), soluble chromatin fragments of 200 to 1000 bp in length were incubated with 5 μg of anti-hERRα antibody, rabbit anti-IgG antibody, or no antibody for 16 h at 4°C, followed by incubation with 80 μg of salmon sperm DNA/protein A-agarose for 2 h at 4°C. Immunoprecipitates were washed and eluted. Samples were then treated with RNase A (Roche Diagnostics) for 30 minutes at 37°C and with proteinase K (Roche Diagnostics) for 2 h at 42°C. Isolated DNA fragments were purified with QIAquick spin kit (Qiagen), and quantitative PCRs were performed using 2 μl of DNA in triplicate. The promoter of FN (−998 to +199) was scanned for putative ERREs using the MAPPER search engine [39]. The primers for four ERRE sites were listed at Supplementary Table S1.

### Animal experiments

Nude mice were purchased from the Sun Yat-sen University (Guangzhou, China) Animal Center and raised under pathogen-free conditions. All animal experiments complied with the Zhongshan School of Medicine Policy on the Care and Use of Laboratory Animals. MDA-MB-231 cells (2 × 10^6^ per mouse) suspended in 100 μl 1640 medium were injected into the fourth right mammary fat pad at the base of the nipple of nude mice (*n* = 9) with 50% Matrigel (BD bioscience, Bedford, MA). When the tumor was visible, mice of XCT-790 group were treated with XCT-790 (4 mg per kg, body weight) by tail vein injection for six times for every three days. When tumor volume at the control group reached approximate 1000 mm^3^, mice were sacrificed, and the tumors were removed and weighed for use in histology and Western blot analysis. Tumor burden in the lung was quantified by manually counting nodules visible on the lung surface. Tumors and lungs were embedded in paraffin for further study.

### Patients and tissue samples

The study included a group of 138 clinical-pathological characterized patients with histologically confirmed triple-negative breast cancer (TNBC) from the Affiliated Cancer Hospital of Guangzhou Medical University and the Cancer Center of Sun Yat-sen University between 2005 to 2014. For all of the patients who participated in this study, written informed consent was obtained, which was approved by the Ethical Committee of Sun Yat-sen University according to the Chinese Ethical Regulations. All samples were tissues collected surgically under the supervision of an experienced pathologist. After collection, samples were stored at −80°C until used. Clinical data were reviewed retrospectively from medical records. The detailed clinical and pathological characteristics of tumor tissues were described in Table 1.

### Tumor histology and immunohistochemistry

Immunohistochemistry staining of formalin-fixed paraffin-embedded tissue was conducted as previously described [40]. Briefly, tumor tissues (both mice and human) and lungs (mice) were fixed in formalin and embedded in paraffin. Sections (5 mm) were cut and stained with H&E. For immunohistochemical staining, sections were deparaffinized and hydrated, and endogenous peroxidase activity was blocked with 3% H_2_O_2_ in water for 10 min. Antigen retrieval was done with 10 mM citrate buffer (pH6.0) for 10 min. Slides were incubated with Biocare blocking reagent for 10 min to block nonspecific binding. Then, they were incubated with anti- ERRα, E-Cad, or FN overnight at 4°C. Slides were washed in PBS twice and then incubated with goat anti-rabbit horseradish peroxidase-conjugated secondary antibodies for 30 min at room temperature and then washed. Finally, slides were incubated with 3, 3′-diami-nobenzidine and counter stained with hematoxylin.

### Statistical analysis

All values were reported as mean ± SD unless otherwise specified. Data were analyzed by two-tailed unpaired Student's *t*-test between two groups and by One-Way ANOVA followed by Bonferroni test for multiple comparison involved. The χ^2^ test or Fisher's exact test was used to analyze the association of ERRα expression and clinical-pathological parameters or ERRα and FN expression in clinical tissues. The survival curves were plotted by using Kaplan–Meier analysis. Statistical analysis was carried out using SPSS 16.0 for Windows. A *p*-value of < 0.05 was considered to be statistically significant.

## SUPPLEMENTARY FIGURES AND TABLE


