# Supplementary material for: Inhibition of ERRα suppresses epithelial mesenchymal transition of triple negative breast cancer cells by directly targeting fibronectin
Source: Oncotarget. 2015 Jun 26;6(28):25588–601. doi: 10.18632/oncotarget.4436 (PMC4694852; doi:10.18632/oncotarget.4436)
Supplement: Supplementary file 1 [file oncotarget-06-25588-s001.pdf]

SUPPLEMENTARY FIGURES AND TABLE

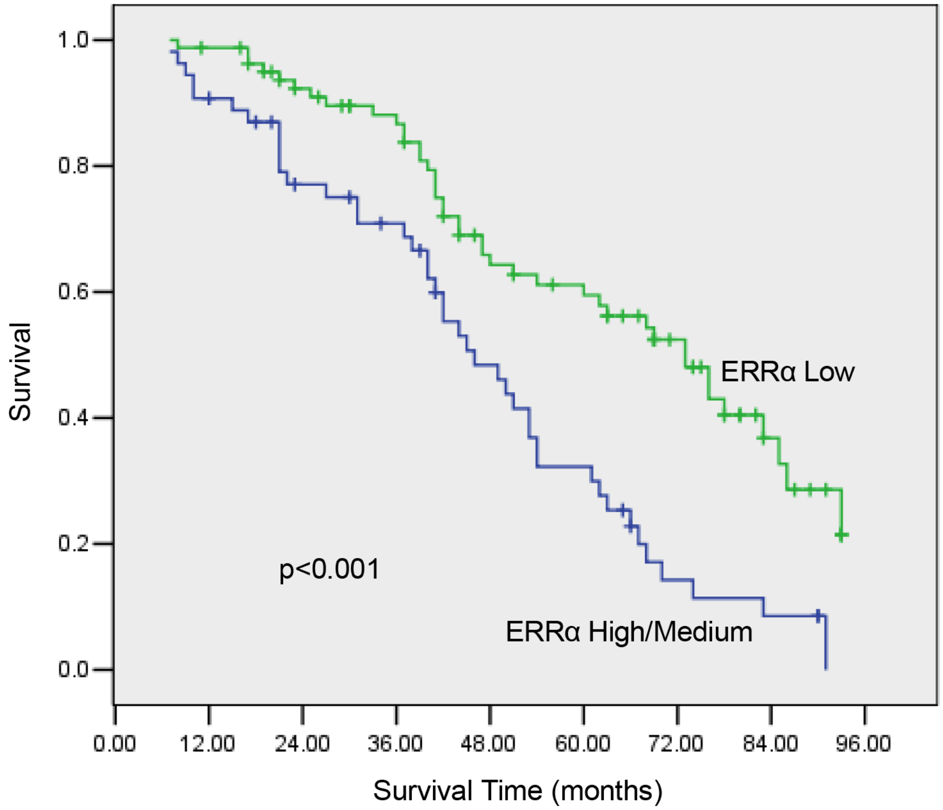

Supplementary Figure S1: ERRα expression is negatively correlated with prognosis in TNBC patients. Overall survival (OS) in patients with high/medium levels of ERRα (*n* = 64) *vs* the remaining patients (*n* = 74) was plotted by the Kaplan-Meier method. Statistical comparison of survival between groups with the log-rank statistic analysis suggests that patients whose tumors express increased levels of ERRα had poorer survival compared with those with low levels of ERRα ($p < 0.001$).

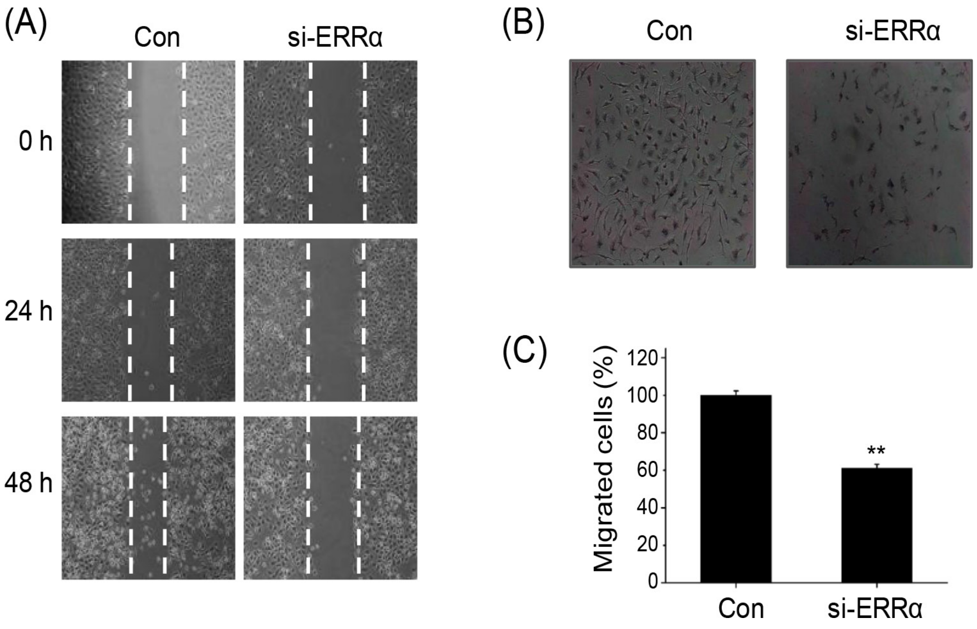

Supplementary Figure S2: ERRα knockdown inhibits the *in vitro* motility of MDA-MB-231 cells. A. MDA-MB-231 cells were transfected with si-NC (negative control) or si-ERRα, and then confluent monolayers of MDA-MB-231 cells were scraped by a pipette tip to generate wounds and observed for the indicated times. MDA-MB-231 cells were transfected with si-NC or si-ERRα and then allowed to migrate transwell chambers for 48 h. The migrated cells were fixed, stained, photographed **B.** and quantified **C.** Data represent the average of three independent experiments, $^{**}p < 0.01$.

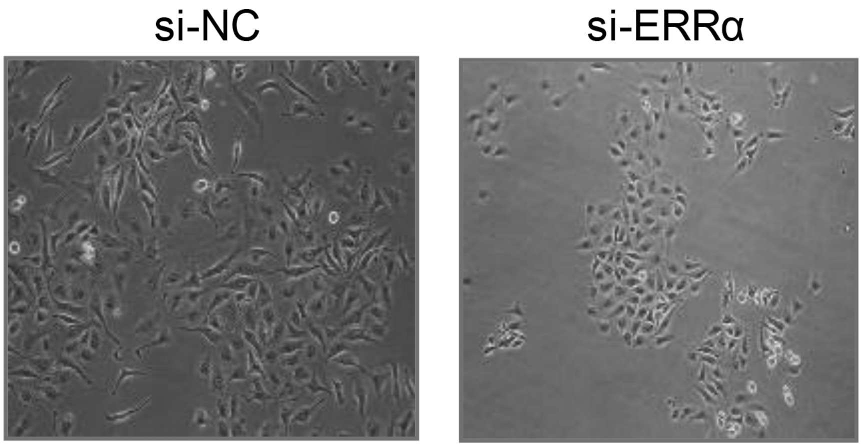

Supplementary Figure S3: The cellular morphology of MDA-MB-231 cells transfected with si-NC or si-ERRα for 24 h.

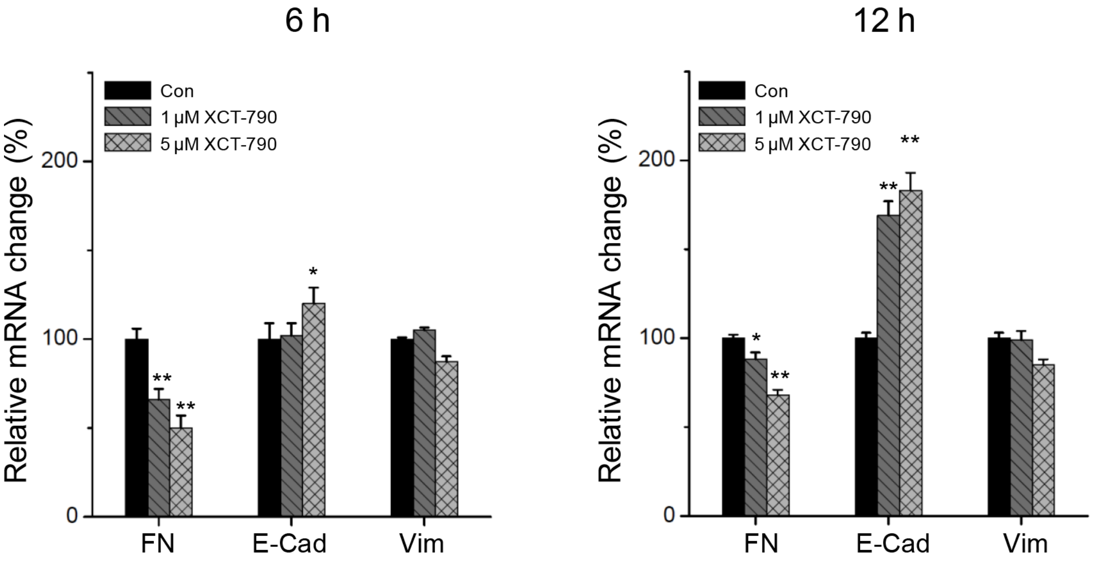

Supplementary Figure S4: Effects of XCT-790 on the mRNA levels of EMT-related markers in BT-549 cells. BT-549 were treated with or without XCT-790 for 6 or 12 h, and then the mRNA levels of FN, E-Cad, and Vim were measured by use of real-time PCR. Data represent the average of three independent experiments, $**p < 0.01$.

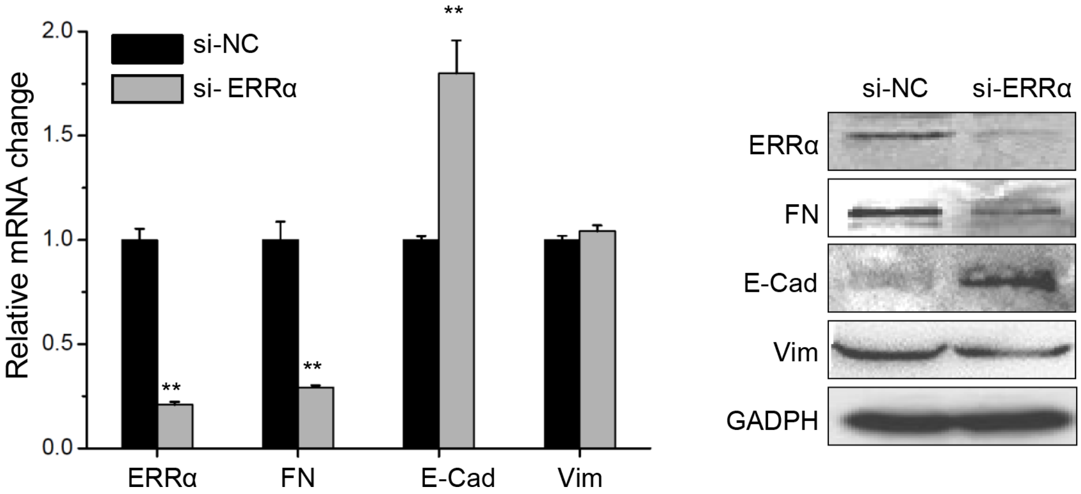

Supplementary Figure S5: Effects of si-ERRα on the mRNA and protein levels of EMT-related markers in MDA-MB-231 cells. MDA-MB-231 cells were transfected with si-NC or si-ERRα for 24 h, and then the mRNA levels of ERRα, FN, E-Cad, and Vim were measured by use of real-time PCR. After transfection for 48 h, the protein levels of ERRα, FN, E-Cad, and Vim were measured by Western blot analysis. Data represent the average of three independent experiments, $**p < 0.01$.

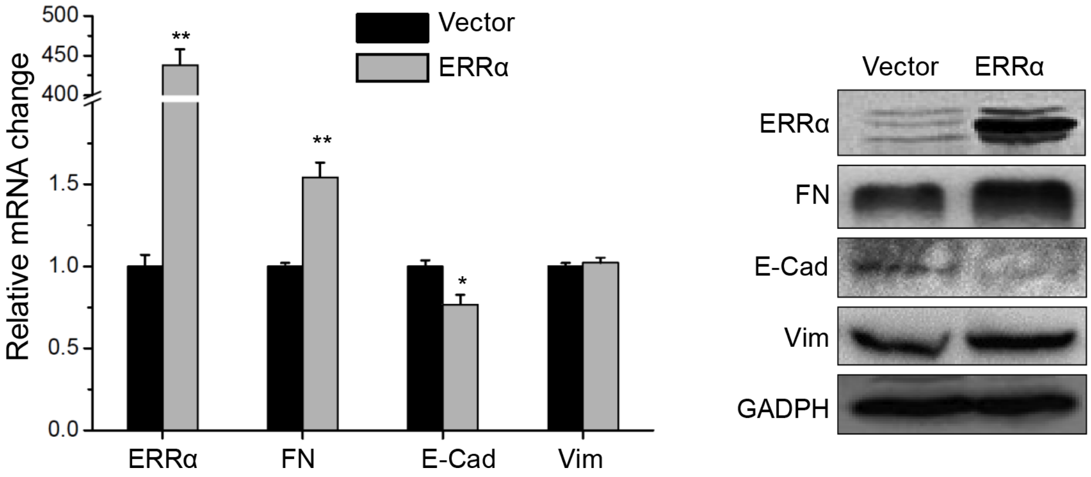

Supplementary Figure S6: Effects of over expression of ERRα on the mRNA and protein levels of EMT-related markers in MDA-MB-231 cells. MDA-MB-231 cells were transfected with pcDNA3.1 or ERRα construct for 24 h, and then the mRNA levels of ERRα, FN, E-Cad, and Vim were measured by use of real-time PCR. After transfection for 48 h, the protein levels of ERRα, FN, E-Cad, and Vim were measured by Western blot analysis. Data represent the average of three independent experiments, *$p < 0.05$, **$p < 0.01$.

Supplementary Table S1: The sequence of primers for the four ERREs measurement during ChIP analysis

	Forward (5′ to 3′)	Reverse (5′ to 3′)	Length
ERRE-1	AGGACATTGCGTCACCTCTCTT	TCCCGAGTCAGTACCCTTTAGTC	196
ERRE-2	CACAGCTCCCTGTTCGGACTTCTT	CGCGGAACTCCCGGTACTTAGTAG	136
ERRE-3	GTGAAATCACTGTGAACAACCTGAA	AAATCTCTGCTTTGTGTGTAACTGG	115
ERRE-4	GTGTTAGGAGTTCAGAGGTTCC	AATGCTAATTTTGTGTCTTTAATGT	180
